# EHMTI-0339. Pain 101: managing complex chronic headaches across the lifespan

**DOI:** 10.1186/1129-2377-15-S1-D15

**Published:** 2014-09-18

**Authors:** B Dick, M Simmonds, S Rashiq, K Reid, M Verrier

**Affiliations:** 1 Anesthesiology and Pain Medicine, University of Alberta, Edmonton, Canada; 2 Pediatric Chronic Pain Clinic, Stollery Children's Hospital, Edmonton, Canada

## Introduction

Headache pain has been found to be a common pain complaint across cultures. Much remains to be learned regarding the etiology and biological treatments of complex headaches. Much is unknown regarding etiology and biological treatments of headache

## Aim

To examine the efficacy of a best practice psychological treatment program based on 3rd wave cognitive- behaviour therapy incorporating Mindfulness-Based Stress Reduction in adolescents and adults with complex headaches.

## Method

Outpatients referred to Stollery Children’s Hospital’s Pediatric Chronic Pain Clinic and University of Alberta Hospital Multidisciplinary Pain Centre were enrolled in the Pain 101 pain management program. Of these individuals, 52 adolescents and 139 adults reported complex headache. Pain 101 uses 10 psychoeducational group sessions aimed at the key areas of pain education, reducing physiological arousal, goal setting and activity management, cognitive reappraisal and acceptance, and emotional regulation. Data collection is targeted for five years post-assessment.

## Results

Headache sufferers across the lifespan showed significant reductions in pain, pain-related disability, anxiety, pain-related fear, and a significant improvement in sleep and quality of life. Adults but not pediatric patients showed depression reduction. In adults, some of these improvements were durable over time to five years post-treatment. Insufficient data is available in the pediatric population at present to reliably discern outcomes past 6-months post-treatment.

## Conclusion

The Pain 101 program shows promise for managing complex headaches and its clinical sequellae. The program successfully reduced disability, improved mood, and increased quality of life in a cost-effective treatment setting. Treatment effects lasted for an extended period post-treatment.

No conflict of interest.

